# Perspectives on technology: All STEPS count – an integrated framework for net zero urological care

**DOI:** 10.1111/bju.16800

**Published:** 2025-06-05

**Authors:** Jamie Hyde, Eleanor King, Joseph John, Kieran O'Flynn, Ian Pearce, John McGrath, William K. Gray, Manraj Phull

**Affiliations:** ^1^ Greener NHS NHS England London UK; ^2^ University of Exeter Medical School Exeter UK; ^3^ Getting It Right First Time (GIRFT) Programme NHS England London UK; ^4^ Royal Devon University Healthcare NHS Foundation Trust Exeter UK; ^5^ Northern Care Alliance NHS Foundation Trust Salford UK; ^6^ British Association of Urological Surgeons London UK; ^7^ Manchester University NHS Foundation Trust Manchester UK; ^8^ University of Bristol Medical School Bristol UK; ^9^ North Bristol NHS Trust Bristol UK

**Keywords:** sustainability, net zero, environment, carbon, emissions, urology, sustainable healthcare

## Abstract

**Objective:**

To present a narrative review of evidence to guide the delivery of high‐quality, low‐carbon urological care using a structured framework.

**Methods:**

Academic and policy papers which outline actions focused on decarbonising urological care and surgical care more broadly were identified and reviewed. The ‘STEPS to Low‐Carbon Care’ framework (an acronym for low‐carbon care across ‘Settings and Treatments, Efficiency, Prevention and System change’) was then used to categorise and present the evidence‐based decarbonisation actions, using the National Health Service in England as a case study.

**Results:**

Across all STEPS framework elements, tangible actions were identified alongside opportunities for future research and innovation. The evidence‐based actions that were identified to transition to low‐carbon care settings and treatments included tackling known carbon hotspots in operating theatres: anaesthetic gases, consumables and electricity use. Outside the operating theatre, urology pathway transformation through one‐stop clinics, day‐case surgery, appropriate use of virtual appointments and streamlined pathways demonstrated opportunities to reduce carbon emissions, with potential additional benefits in terms of cost, efficiencies, and patient outcome improvements. Key climate mitigation actions that support keeping people healthy were identified. There was a paucity of evidence demonstrating the implementation of climate change action as part of routine service delivery. Embedding sustainability across organisational processes and ways of working requires actions to upskill, engage and enable the workforce to deliver and to establish clinical leadership.

**Conclusion:**

This review identified a range of interventions to decarbonise urological care, whilst highlighting a need for further research. Categorising the evidence according to components of the STEPS framework indicated the potential utility of this framework when determining unrealised decarbonisation opportunities in urology and more widely across healthcare. Delivering sustained and system‐wide low‐carbon urological care will require the collective action of all who design, deliver and influence patient care across the specialty and all urology patient pathways.

**Abbreviations**
BAUN: British Association of Urological Nurses
CO_2_e: Carbon dioxide equivalent
GIRFT: Getting It Right First Time
TURBT: Transurethral resection of bladder tumour

## Introduction

Climate change is a global crisis threatening the foundations of good health and the ability of health systems to deliver high‐quality care [1]. The impacts of humans on the environment affect urological diseases and treatments. For example, elevated temperatures are linked to an increased risk of renal tract calculi, and genitourinary cancers have been associated with environmental pollutants or environmental changes [2, 3].

Healthcare contributes approximately 4%–5% of global greenhouse gas emissions, referred to here as ‘carbon’ or ‘emissions’ [4]. Acknowledging this, in 2020, the NHS in England became the first healthcare system to commit to reaching net zero by 2045 [5]. Coordinated by the WHO, 77 countries have committed to building climate‐resilient and low‐carbon health systems, 43 of which currently have net zero targets [6]. Carbon foot‐printing of the NHS in England identified several carbon hotspots to address: the supply chain, including medicines and medical equipment (62% of the NHS's carbon footprint); estates and facilities (15%); travel and transport (14%); and inhalers and anaesthetic gases (5%) [5, 7].

Surgical care has a significant carbon impact, with the United Kingdom's surgical sector responsible for approximately 5.7 million tonnes of carbon dioxide equivalent (CO_2_e) emissions annually [8]. Urological care is a notable contributor, accounting for over 7% of NHS England's surgical spending in 2019/2020 [8]. Tackling urology's carbon emissions is crucial for healthcare decarbonisation given the large clinical activity volumes and the diverse range of conditions treated in this specialty, from recurrent diseases including renal stones and bladder cancer to common disorders including BPH and UTIs. Many urology patients are medically complex; this, coupled with the increasing complexity of oncological treatments, the reliance on advanced imaging technologies, and the use of robotics and of numerous single‐use medical devices, contributes towards the specialty's high carbon emissions.

Delivering low‐carbon care requires a comprehensive approach which navigates the complexities and pressures on healthcare systems, maximises the health, efficiency and cost co‐benefits of climate change mitigation, and maintains or improves patient care. The need to expand sustainability efforts beyond the operating theatre to cover whole patient pathways and consider the wider health determinants is recognised in urology [9] and surgical care more broadly [8].

In this narrative review, we aimed to demonstrate the benefits of a systematic and system‐wide approach by using the low‐carbon care STEPS framework to present our findings. STEPS is a framework for decarbonisation which is applicable across health and social care. It enables consideration of the whole patient pathway, encompassing the breadth of actions and opportunities, seeks to prevent unintended consequences, such as merely reattributing carbon emissions elsewhere in the pathway, and highlights the importance of joined‐up, synergistic action.

## Overview of the STEPS to Low‐Carbon Care: A Systematic Framework for Healthcare Decarbonisation

STEPS is a systematic framework, based on five core principles, for delivering low‐carbon care through consideration of ‘Settings, Treatments, Efficiency, Prevention and System change’.

1. *Settings* encompasses the location of care. This principle focuses on ensuring responsible resource use through efficient use of clinical spaces and energy.
2. *Treatments* is about responsible care practices. It explores ways to optimise treatments (including interventions, medicines and supporting diagnostics), adopt safe and effective low‐carbon alternatives and embed circular economy principles, including the five Rs (Reduce, Reuse, Repair, Remanufacture / Repurpose, and Recycle).
3. *Efficiency* focuses on the delivery of efficient, effective and streamlined care pathways, to ensure timely and appropriate care delivery in the optimal care location.
4. *Prevention* is about keeping people healthy and empowering patients. This principle promotes actions which reduce healthcare's emissions and mitigate the adverse impacts of climate change on health.
5. *System change* is about embedding low‐carbon care into business as usual, namely, into the core functions of the teams, departments and organisations delivering or influencing care. This principle highlights opportunities to integrate sustainability into processes, policies, governance, research, leadership, and workforce training.

The STEPS framework integrates what is known about the carbon footprint of healthcare with known enablers for delivering transformational change in healthcare systems, such as the WHO six pillars of health systems [7, 10], and with established principles for healthcare decarbonisation achieved by taking an end‐to‐end view of the patient pathway [8, 11, 12]. Such holistic approaches highlight the potential mutual benefits of sustainability actions for patients and health systems which include: improving health outcomes, reducing health inequalities, driving healthcare efficiencies and reducing costs [8, 9, 10].

## Methods

**Fig. 1:**
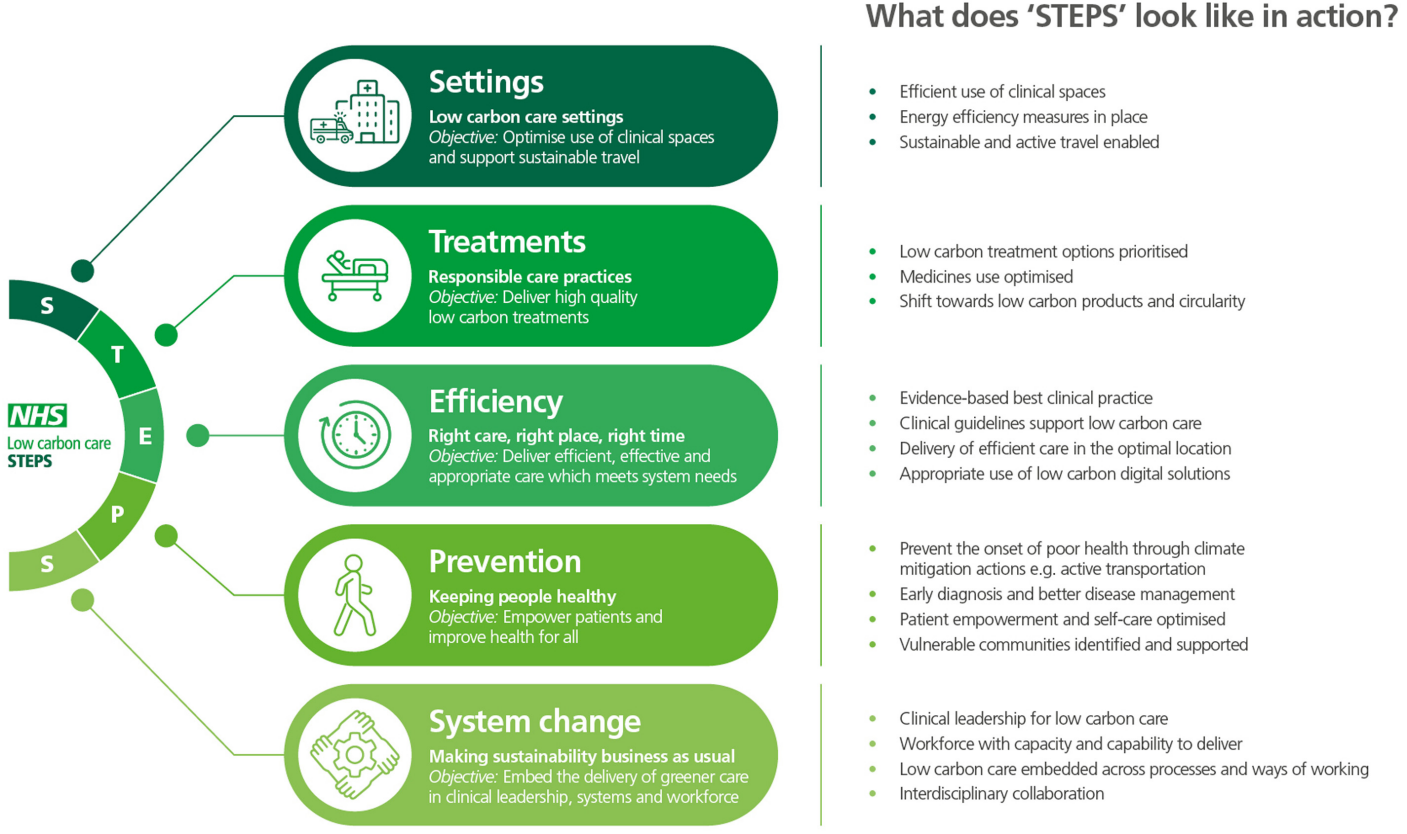
Summary of the Low‐Carbon Care STEPS framework, formed of five guiding principles for delivering high‐quality, equitable, low‐carbon care. This infographic outlines the key details for each of the five ‘STEPS’, including the objective of each one, and the core delivery actions required. While this framework is divided into five key areas for action, there is no requirement to consider or implement the principles in a linear or sequential manner.

For this narrative review we performed a literature search of the Cochrane Library, PubMed, ScienceDirect, Scopus and Google Scholar using the key search terms ‘urology’ or ‘urological care’, in conjunction with ‘carbon’, ‘sustainable’, ‘sustainability’, ‘net zero’ and ‘environment’. We also reviewed the grey literature, looking at key papers or policy documents from government, think tanks and professional bodies. Titles and abstracts were screened according to relevance to the topic and reliability, as determined by three authors (M.P., J.H., E.K.). A total of 83 full‐text papers were reviewed, including 13 peer‐reviewed primary research papers and two peer‐reviewed meta‐analyses. Papers produced unilaterally by industry and non‐peer‐reviewed case studies were excluded. Study outcomes were explored narratively and categorised using the STEPS to low‐carbon care framework and key actions (Fig. 1). This also facilitated identification of knowledge gaps, which are explored within this review.

### **S**ettings: Low‐Carbon Care Settings

**Table:** 

**Key STEPS Decarbonisation Mechanisms Applied to Urological Care** Maximising use of low‐resource, energy‐efficient care settings. Improving energy efficiency through behaviour change. Embedding energy efficiency into infrastructure. **Existing Evidence Demonstrating Application in Urology** Operating theatres are three to six times more energy‐intensive than wards, emphasising the need for energy efficiency in these high‐consumption areas [8]. Flexible cystoscopy and bladder tumour ablation in a clinic setting to reduce the reliance on energy‐intensive operating theatres [13, 14, 15, 16]. New BPH treatment technologies that can be delivered as day‐case procedures, outside operating theatres [15]. Outpatient‐based local anaesthetic prostate biopsies [17].

*Maximising use of low‐resource, energy‐efficient care settings*, without compromising patient safety or outcomes, can significantly reduce carbon emissions [8]. ‘Low‐resource’ refers to settings associated with low use of energy and consumables, and with relatively low reliance on infrastructure or equipment. The Getting It Right First Time (GIRFT) programme demonstrates the benefits of moving certain surgical procedures from theatres to alternative settings [14]. The feasibility of clinic‐based treatments has been established in several procedures to date, such as flexible cystoscopy and bladder tumour ablation [13, 14, 16]. Additionally, GIRFT has demonstrated that local anaesthetic transperineal prostate biopsy can be routinely delivered in a clinic setting, avoiding the use of resource‐intensive operating theatre settings; technology that optimises theatre time and use also supports this goal [14, 15, 16, 17].

*Improving energy efficiency through behaviour change* to switch off equipment when not in use – such as anaesthetic gas scavenging systems, air handling units, and computers – can deliver significant carbon and financial savings. Switching ventilation systems off overnight instead of using low‐power mode saved one UK hospital £30 000 annually per operating theatre [8]. The behaviour change initiative ‘Operation TLC’ (Turn off equipment, Lights out, Control temperatures) saved 2200 tCO_2_e and £0.5m in 1 year at one NHS trust, with projected annual reductions of £35m and 155 ktCO_2_e if implemented across all trusts nationally [18, 19]. Shutdown checklists have been shown to be effective in prompting equipment switch‐off in theatres [8], and the Intercollegiate Green Theatre Checklist covers a range of actions implementable within theatres to reduce operative resource use [20]. Implementing energy efficiency measures outside of operating theatres in areas such as inpatient wards and outpatient clinics is also important [9].

*Embedding energy efficiency into infrastructure* can be achieved when developing new facilities or refurbishing existing spaces, particularly in operating theatre complex design. Installing automated lighting, temperature and ventilation controls can support energy reduction in theatres, alongside procurement of energy‐efficient appliances, computers and equipment. Aligning with net zero building standards ensures energy‐efficient choices become the default in healthcare facilities, and procuring renewable energy supports reduced emissions [21].

### **T**reatments: Responsible Care Practices

**Table:** 

**Key STEPS Decarbonisation Mechanisms Applied to Urological Care** Implementing known low‐carbon treatment alternatives. Identifying and prioritising low‐carbon treatments. Applying sustainable procurement principles and the five Rs (Reduce, Reuse, Repair, Remanufacture / Repurpose, and Recycle). Optimising medicine use, including anaesthesia. **Existing Evidence Demonstrating Application in Urology** Low‐carbon anaesthetic techniques, including spinal and local anaesthesia, can be used instead of general anaesthesia for many urological procedures [8]. Over 60% of healthcare emissions are from procured goods [5]. In surgery some studies estimate 20% of emissions are from procured goods, noting this is ‘underestimated’, as Scope 3 emissions are not included [11], and others estimate over 80% [13]. Transurethral resection of bladder tumour (TURBT) peri‐operative pathways highlight anaesthesia and equipment as key carbon hotspots [13]. Switching from single‐use to reusable cystoscopes has shown an overall carbon reduction per case [8].

*Implementing known low‐carbon treatment alternatives* can improve patient outcomes and reduce emissions. ‘Treatments’ here refers to medical and surgical interventions and any supporting diagnostics. Known solutions exist, for example, in anaesthesia. The NHS‐wide decommissioning of desflurane, a carbon‐intensive anaesthetic gas, has already reduced carbon in urological surgeries [22]. Day‐case surgery has been shown to reduce attributable carbon emissions for certain urological operations by avoiding hospital admission [13, 15].

With growing emphasis on day‐case surgeries and the benefits of avoiding general anaesthesia for the increasingly frail and elderly urological patient population, spinal and local anaesthesia are increasingly used. These anaesthetic techniques are associated with lower carbon emissions compared to general anaesthesia, either through avoiding release of volatile anaesthetic agents, or through reducing the single‐use plastic and airway equipment used in total intravenous anaesthesia. The anaesthetic community broadly agrees on total intravenous anaesthesia as a preferred technique over volatile anaesthesia on environmental grounds (64%–68% of anaesthetists surveyed), with a need for more education and information to reassure anaesthetists of its safety and benefits [23]. Another priority is reducing nitrous oxide waste. Nitrous oxide accounts for almost 2% of NHS emissions [5]. System leaks, stock management issues and oversupply have resulted in the waste of nearly 100% of piped nitrous oxide in some settings [24]. Decommissioning centrally piped systems in favour of portable cylinders is a high priority and will provide significant environmental and financial benefits [24].

*Identifying and prioritising low‐carbon treatments* involves reviewing the practices, products and treatments used in urological care pathways. This can range from pharmacotherapy to major surgery. Care pathway‐level carbon impact assessment is an emerging field, with studies differing in their levels of detail and methodological choices.

The foremost example of implementation of low‐carbon treatments to date is in anaesthesia, where key enablers have included strong clinical leadership to champion interventions alongside proactive action and multidisciplinary collaboration from frontline staff [23]. This has also been aided by having a well‐defined problem and available alternative treatments (such as different inhalational anaesthetic agents, total intravenous anaesthesia, and regional anaesthesia) offering equivalent therapeutic effect. As the field of carbon footprinting surgical approaches is nascent, like‐for‐like comparisons between treatment options should be considered with caution. For example, robot‐assisted surgery might commonly have higher emissions associated with surgical equipment use compared with open surgery, but at a care pathway level the picture is less clear. Evidence indicates that open cystectomy and prostatectomy are both associated with longer lengths of stay, more transfusions and slower convalescence – for cystectomy this difference is observed over months [25, 26, 27, 28]. Taking this wider view of healthcare resource utilisation, it is quite possible that emissions for patients undergoing open surgery might be greater, on average, than for patients undergoing robot‐assisted surgery, although there is a paucity of evidence to support this conclusion currently. Regardless, a finding in favour of the environmental performance of robot‐assisted surgery would not negate the need for robot‐assisted surgical equipment manufacturers to take all reasonable steps to reduce the emissions related to their products. This includes addressing avoidable planned obsolescence and over‐reliance of single‐use plastics.

*Practising sustainable procurement and applying the five Rs* supports reducing the 62% of NHS carbon emissions associated with procured goods and services [5, 7]. Medical and surgical equipment accounts for 10% of all NHS emissions [8] – a major carbon hotspot also identified in the TURBT peri‐operative pathway [13]. Meeting the NHS commitments outlined in the NHS Net Zero Supplier Roadmap [29] and embedding sustainability in procurement decision making requires action across the urological and wider multidisciplinary team.

Prioritising changes in procurement and product usage can initially focus on the numerous high‐impact, readily achievable actions that do not compromise clinical care, before addressing more complex challenges. The Green Surgery Report [8] outlines approaches such as shifting from ‘just in case’ to ‘open when required’, which have shown measurable carbon and cost reductions, for example, biopsy forceps or Ellik evacuators [13]. In urology, consolidating reusable surgical equipment sets into smaller or fewer sets can substantially reduce reprocessing‐related carbon emissions [30].

Reducing routine use of postoperative urethral catheters and avoiding unnecessary postoperative irrigation and catheterisation supports emissions reduction. Irrigation fluid management and related plastic waste in urological surgeries are carbon hotspots [31]. Optimising intra‐operative irrigation flow and reducing reliance on postoperative irrigation by achieving good haemostasis and delivering day‐case endourology surgery are key priorities for reducing irrigation‐related carbon emissions. The method of intra‐operative irrigation disposal is also important; one hospital estimated avoiding 3203 kgCO_2_e carbon emissions over 8 months using a direct‐to‐drain fluid management system instead of using plastic suction liners that are incinerated [31].

Debate over single‐use vs reusable medical equipment in surgery is driven by a lack of rigorous product‐level carbon footprinting – the Green Surgery Report reviews the evidence available, concluding that average carbon savings of 38%–56% can be achieved through switching to reusable equipment [8]. Use of reusable cystoscopes has been shown in one US study to reduce the carbon footprint by 22% per case compared to single‐use cystoscopes [32].

Incineration costs for clinical waste in the NHS in England exceed £70m annually. Improving urological waste management systems through simple measures, such as introducing recycling bags and staff training, helped recycle 1.85 tonnes of plastic waste from urology services in 1 year, avoiding incineration at one hospital site in England [31]. Combining waste from multiple patients' treatments can reduce the total volume of hazardous waste incinerated [13].

In future, re‐adopting reusable intermittent self‐catheters may support substantial reduction of the carbon emissions associated with management of chronic urinary retention, as part of a carefully considered move back from reliance on the linear economy. Historically, patients routinely used reusable self‐catheters. Although adoption of single‐use catheters has risen, these have not shown significant benefit over reusable versions regarding infection rates in intermittent catheterisation and are likely to have much higher associated carbon emissions [33].

*Optimising medicine use* can improve care whilst reducing waste and carbon emissions. Approximately 10% of prescribed medicines are wasted in England, accounting for 202 tCO_2_e per 100 000 residents [34, 35]. Careful antibiotic stewardship, reduced polypharmacy and discontinuing ineffective medications are key to addressing this [34, 35]. Additionally, there is evidence and guidance to support the use of more conservative interventions in the first instance. For example, the TRIUMPH study, a 2023 randomised controlled trial across 30 general practice sites in England, demonstrated sustained reductions in LUTS in men treated conservatively using standardised information leaflets and guidance on lifestyle interventions in primary care [36]. This avoided the need for medical management altogether for a subset of men.

### **E**fficiency: Right Care, Right Place, Right Time

**Table:** 

**Key STEPS Decarbonisation Mechanisms Applied to Urological Care** Streamlining care pathways to reduce unnecessary appointments, procedures and follow‐ups through same‐day or one‐stop appointments, appropriate discharge, and patient‐initiated follow‐up. Shifting care to community and home settings where appropriate, using digital technologies to reduce patient travel and optimise care delivery. Optimising imaging and pathology testing. **Existing Evidence Demonstrating Application in Urology** Same‐day preoperative assessment clinics reduce patient travel and emissions [13]. Day‐case TURBT procedures can have a lower carbon footprint by avoiding inpatient care [37]. Virtual clinics have been estimated to reduce carbon emissions by up to 99% in one study [38]. Ultrasound imaging has a significantly lower energy use per min (23 Wh) compared to CT (939 Wh) and MRI (605 Wh) and a lower estimated total carbon footprint per scan performed [39].

*Streamlining care pathways* to reduce unnecessary appointments, duplicative tests, lengthy inpatient stays and avoidable patient journeys improves patient experience and reduces carbon emissions. For example, urological one‐stop clinics combine consultations, tests and minor procedures into one visit, avoiding multiple trips and associated emissions, while improving patient satisfaction. An 18% increase in day‐case TURBTs over 8 years was estimated to have avoided 2900 tCO_2_e of carbon emissions in England. If all NHS hospitals in England achieved a 51% day‐case surgery rate, 372 tCO_2_e could be saved annually compared with average practice observed in 2021/2022 [37].

*Shifting care to community and home settings* where appropriate can reduce unnecessary inpatient stays and patient travel, reducing carbon at the patient level as well as potentially improving patient care and health system operational capacity. In urology, solutions include optimising use of primary and community care to manage long‐term conditions, such as prostate cancer monitoring and long‐term catheter care, and maximising use of community diagnostic hubs to offer care closer to home. Virtual clinics can reduce carbon emissions by up to 99% compared to face‐to‐face appointments, through minimising patient travel [38]. Teleconsultations for circumcision assessments during the first COVID‐19 lockdown were found to be as effective as in‐person visits and avoided 637 kgCO_2_e for every 100 patients [40].

Digital technologies can enable virtual and remote care, presenting opportunities to reduce emissions. While most studies focus on changes to travel‐related carbon from digitally enabled care, the environmental impact of digital hardware, energy consumption and data storage warrants consideration. Initiatives aiming to mitigate these emissions include enabling device reuse, minimising energy from device idling and optimising energy sources [41]. Digital literacy and digital inclusion are key factors when considering widespread adoption of virtual care [42].

*Optimising imaging and pathology testing* are priorities, given their frequent use, high energy consumption and reliance on single‐use items. The NHS performs approximately 40 million radiological investigations and 800 million laboratory tests annually, with up to 40% of the latter deemed clinically unnecessary [43, 44]. Clinicians can improve environmental, financial and patient outcomes by choosing the most appropriate diagnostic modality, reducing avoidable tests and choosing lower‐carbon imaging options. MRI and biopsy pathways for prostate cancer are particularly carbon‐intensive, energy use being the main contributor to emissions [39]. Key strategies to reduce these emissions include using low‐carbon electricity and considering adopting biparametric instead of multiparametric prostate MRI; this is shorter, avoids contrast and cannulation, and has been shown to be non‐inferior in a recent randomised controlled trial [45].

### **P**revention: Keeping People Healthy

**Table:** 

**Key STEPS Decarbonisation Mechanisms Applied to Urological Care** Promoting active lifestyles and low‐carbon diets – which are low in red meat – to reduce carbon emissions, prevent disease and improve recovery from surgery. Reducing travel emissions through remote care, active travel, and low‐carbon transport options. Empowering patients to manage their health, reducing hospital visits and emissions. **Existing Evidence Demonstrating Application in Urology** Obesity increases surgical complexity and prevalence of certain urological conditions such as urolithiasis [46, 47]. Approximately 19% of the peri‐operative TURBT pathway emissions come from travel according to one study, highlighting the need to reduce unnecessary patient journeys [13]. Self‐removal of ureteric stents can empower patients while reducing hospital visits and travel emissions, with 90% of patients preferring this option [48]. Similar evidence is emerging for self‐trial without catheter [49].

*Promoting healthier, low‐carbon diets and active lifestyles* is an opportunity to improve population health and reduce health systems' carbon emissions both directly and indirectly. Rising obesity rates lead to higher surgical complexity, longer operations and more postoperative complications [46]. Obesity contributes to oxalate and uric acid stone formation in the upper urinary tract, a significant risk factor in urological conditions such as urolithiasis [47]. Effective health promotion, including staff and patient education on the benefits of healthier diets and empowerment to make positive changes within healthcare systems, can reduce surgical risk factors and potentially lower overall environmental impact [50]. Red meat in particular increases salt and saturated fat intake, increasing the risk of urological cancers, and is associated with higher carbon emissions [51]. Practically, offering patients and healthcare staff healthier, low‐carbon food options, which are more plant‐based and use more sustainable seasonal and local fruits and vegetables, also supports this. Urologists are well placed to provide dietary and fluid intake advice to patients with urolithiasis and renal impairment, which often aligns with low‐carbon food choices [52]. Low‐carbon nutritional provision involves minimising food waste, requiring collaboration with catering teams [5].

*Reducing travel emissions through remote care, active travel, and low‐carbon transport options* benefits patients and the environment. Research attributes approximately 19% of peri‐operative TURBT pathway emissions to patient and visitor travel [13]. To reduce these emissions, care access can be reconfigured to minimise unnecessary travel. This includes utilising one‐stop approaches and digital communications where appropriate, for example, to support remote consultations. When travel is necessary, encouraging active travel (walking, cycling, wheeling) or public transport options can further reduce carbon emissions. Additionally, supporting staff to use low‐carbon transport options, including electric vehicles and car sharing, improves local air quality and overall population health by reducing particulate emission concentrations [53].

*Empowering patients* to take an active role in managing their health, including through education, can lead to improved disease management, earlier detection of health conditions, and prevention of avoidable hospital visits. This enables more community or home‐based care, which can improve patient satisfaction, reduce travel emissions and optimise hospital resource use. One study found that 90% of patients preferred the self‐removal of ureteric stents using a stent‐on‐strings, with 60% finding the process ‘very easy’. Home‐based stent‐on‐strings removal reduces follow‐up hospital visits and can enable financial savings [48]. Additionally, a recent study has demonstrated the carbon savings potential of self‐trial without catheter after robot‐assisted radical prostatectomy [49].

### **S**ystem Change: Making Sustainability Business‐as‐Usual

**Table:** 

**Key STEPS Decarbonisation Mechanisms Applied to Urological Care** Supporting clinical leadership and workforce training in low‐carbon care. Embedding sustainability into urology‐specific healthcare operations, governance, standards and policies. Promoting research and innovation in sustainable urological practices. **Existing Evidence Demonstrating Application in Urology** Leadership from all relevant healthcare professionals in urological surgery, including surgeons, anaesthetists, nurses, and estates managers, is crucial for implementing low‐carbon interventions across urological care pathways [54]. The BAUS is incorporating sustainability into audits to monitor progress on sustainable clinical transformation, available through the BAUS website (baus.org.uk). Clinical trials account for an estimated 27.5 MtCO_2_e emissions, highlighting the need for research and innovation to address the carbon footprint of clinical trials [55].

*Supporting clinical leadership and workforce engagement* are underpinning enablers of low‐carbon healthcare as identified by the Health Foundation, alongside supportive policy levers and accelerated research and innovation [56]. A 2021 YouGov survey found that nearly 9/10 NHS staff support net zero, with many already pursuing environmental sustainability. However, workplace carbon literacy remains inconsistent, with insufficient leadership in environmentally sustainable healthcare delivery hindering decarbonisation of surgical care [54]. Clinical leaders must be enabled to champion and support the delivery of low‐carbon interventions actively at all levels, from professional and representative organisations to individual departments and frontline staff. Developing and providing access to education is crucial. BAUS's recent plenary session on sustainability shows the potential in this area and provides momentum for the specialty. The Intercollegiate Green Theatre checklist provides practical guidance to reduce the environmental impact of surgical procedures [20]. Such support enables individuals to contribute to healthcare's sustainability goals. Coordinated collaboration in urology – among surgeons, nurses, anaesthetists, allied health professionals, patients, and estates managers – can harness these multidisciplinary groups' diverse expertise to design and deliver low‐carbon urological pathways. Training and education on low‐carbon healthcare can foster positive action. However, a 2022 survey revealed that 7/10 surgical staff had not received any sustainable healthcare education [57]. The General Medical Council now requires inclusion of sustainable healthcare education in medical curricula, and further efforts to incorporate sustainability into undergraduate and postgraduate medical training would be welcomed [58].

*Embedding sustainability into urology‐specific healthcare operations, governance, standards and policies* sets the foundations for lasting system change. Integrating meaningful evaluation of sustainability goals into existing mechanisms, such as quality improvement initiatives, can reduce carbon and improve other key outcomes including patient safety, efficiency and equity [12]. All GIRFT academy guidance, co‐developed with BAUS, the British Association of Urological Nurses (BAUN) and other specialty associations, is now to incorporate guidance on sustainability [59]. The recent publication of GIRFT's first greener pathway demonstrates this in practice. The new green pathway guide developed in conjunction with Greener NHS, BAUS, BAUN, the Royal College of Surgeons of England, and the British Association of Day Surgery outlines practical steps towards decarbonising the bladder cancer pathway, helping to reduce the environmental impact of the NHS while improving patient care. The guide lists 12 high‐impact clinical recommendations for decarbonising bladder cancer care, alongside the potential annual emissions reduction across England if the recommendations are carried out [59]. Building on this, BAUS will conduct mandated national audits in England through NHS quality accounts to evaluate progress on sustainability, available for BAUS members through BAUS (baus.org.uk).

*Promoting research and innovation in sustainable urological practices* is crucial for the transition to net zero urology. Expanding the evidence base to inform policies and the development of new, sustainable products and procedures is essential to overcome perceived barriers to low‐carbon surgical care, for instance, infection prevention and control practices. Decarbonising clinical research is also necessary, as clinical trials alone contribute an estimated 27.5 MtCO_2_e across healthcare [55]. Innovation funding through the Small Business Research Initiative is a promising step, supporting the development of novel decontamination methods, reusable products and non‐sterile alternatives [60]. Cross‐sector collaboration facilitates effective horizon scanning, demand‐signalling and the rapid uptake of solutions. This can also support wider action to influence the structural determinants of health and tackle both decarbonisation and health inequalities.

### Research Gaps and Priorities

We have identified research gaps and priorities across several common themes.

#### Lack of Pathway‐Level Studies

Studies are primarily narrow in scope, looking at individual products or treatments. Few have taken a broader view across patient pathways. A high proportion of existing research focuses on a small number of outcomes and variables, such as either cost or carbon reductions, making it difficult to draw conclusions about the overall effects within a patient pathway or at a system level. Researchers should consider evaluating a broader range of outcome measures including clinical, environmental and financial outcomes, alongside carbon and additional environmental effects.

#### Methodological Limitations among Product‐Level Environmental Assessments

Academic papers and reports that assess the carbon emissions of individual products often use very different methodologies or have significant methodological limitations including a lack of transparency, assumptions, and limited modelling of different usage scenarios [61]. Many papers favouring single‐use items [62, 63] offer limited methodological detail on how comparisons have been made, so findings are hard to interpret objectively. Consequently, understanding the potential benefits of reusable equipment can be more challenging in urology than in other specialties. The Green Surgery Report provides the closest to a comprehensive review of the issue and endorses the use of reusable equipment where clinically appropriate [8]. The prevailing findings from methodologically robust studies indicate that adherence to circular economy principles will typically result in lower carbon emissions, although we should always consider reporting bias.

#### Lack of Investigation into Ongoing Care, Long‐Term Care, and Prevention

Most papers focus on discrete parts in the early stages of urology pathways, covering assessments and diagnostics, initial treatment and follow‐up. Few consider ongoing care, discharge, primary or secondary prevention and system‐level interventions. A key future research priority should therefore be to address these additional areas as part of low‐carbon, high‐quality, whole care pathway design.

## Conclusion

Using the STEPS framework, we have outlined the different evidence sources demonstrating how routine urological care can be transformed to deliver a service with lower carbon emissions, whilst also meeting the urological health needs of our population. We have outlined various potential co‐benefits associated with targeting low‐carbon urological care, including improved clinical outcomes and patient experience, lower costs, and improved health service efficiency. Rapidly and substantially decarbonising a complex health system is itself a highly complex task, and the STEPS framework can function as a useful tool to guide further low‐carbon research, innovation, and implementation within urology and across healthcare.

## Disclosure of Interests

The authors have no disclosures.
